# Clinical Performance of ChatGPT in Diagnosing Degenerative Diseases: A Case‐Based Assessment

**DOI:** 10.1002/alz70857_101996

**Published:** 2025-12-25

**Authors:** Pan‐Woo Ko

**Affiliations:** ^1^ School of Medicine, Kyungpook National University, Daegu, DAEGU, Korea, Republic of (South)

## Abstract

**Background:**

With the advent of AI tools like ChatGPT, evaluations of their usefulness are being conducted across various professional fields, including medicine. In our previous study utilizing ChatGPT version 3.5, we assessed its performance on in‐service exam questions from neurology residency training. Out of 113 questions, ChatGPT correctly answered 51, achieving an accuracy rate of 45.1%. This result was interpreted as corresponding to the knowledge level of a first‐year resident when compared to annual exam results. The accuracy varied greatly across sub‐specialties, ranging from 0% in neuro‐ophthalmology to 80% in sleep medicine. The present study aims to evaluate the clinical performance of ChatGPT in the context of degenerative diseases.

**Method:**

To maintain consistency, we used nine case reports from the “Letter to the Editor” section published in the journal ‘*Dementia and Neurocognitive Disorders’* from 2022 to 2024. Initially, we input the clinical information including descriptions of patient symptoms, medical history, and neurological examination findings and asked ChatGPT to provide differential diagnoses and appropriate diagnostic methods. Following this, we input the test results and asked for the final diagnosis. The ChatGPT version 3.5 was used for the evaluation.

**Result:**

In the first round of questioning, ChatGPT included the correct final diagnosis in its differential list for 3 out of the 9 cases (accuracy rate 33.3%). For the appropriate diagnostic methods as outlined in the case reports, ChatGPT correctly suggested them in 8 out of 9 cases (accuracy rate 88.9%). In the second round, when asked for the final diagnosis, ChatGPT's suggested diagnosis matched the case's final diagnosis in 7 out of 9 cases (accuracy rate 77.8%).

**Conclusion:**

This study demonstrated that AI tools like ChatGPT exhibit strong performance in assessing clinical tasks related to dementia, based on case reports published in specialized journals. While there are limitations to text‐based questioning and the structured format of journal reports, ongoing advancements in AI technology offer promising prospects for improved performance and broader applications in the dementia field.

